# Psychiatry Trainees' Attitudes, Knowledge, and Training in Addiction Psychiatry—A European Survey

**DOI:** 10.3389/fpsyt.2020.585607

**Published:** 2021-01-08

**Authors:** Laura Orsolini, Irena Rojnić Palavra, Gabriele Duccio Papanti, Matej Potočan, Diego Quattrone, Matis Martens, Sandra Sklenářová, Jonna Levola, Leslie Grichy, Sean Naughton, Indre Kotryna Grinevičiene, Jelly Petra Kuiters, Tomasz M. Gondek, Anca-Livia Panfil, Milica M. Borovcanin, Alberto San Roman Uria, Ewelina Biskup, Ekin Sönmez Güngör, Marisa Casanova Dias, Sonila Tomori, Visnja Banjac, Petra Marinova-Djambazova, Mariana Pinto da Costa

**Affiliations:** ^1^Unit of Clinical Psychiatry, Department of Clinical Neurosciences/DIMSC, School of Medicine, Polytechnic University of Marche, Ancona, Italy; ^2^Psychopharmacology, Drug Misuse and Novel Psychoactive Substances Research Unit, School of Life and Medical Sciences, University of Hertfordshire, Hatfield, United Kingdom; ^3^Psychiatric Hospital Sveti Ivan, Zagreb, Croatia; ^4^Mental Health Department, Friuli Venezia Giulia, Italy; ^5^Psychiatric Hospital Begunje, Begunje na Gorenjskem, Slovenia; ^6^Social, Genetic and Developmental Psychiatry Centre, Institute of Psychiatry, Psychology and Neuroscience, King's College London, London, United Kingdom; ^7^Vaasa Central Hospital, Psychiatry Outpatient Clinic, Vaasa, Finland; ^8^Department of Child Psychiatry, Charles University Second Faculty of Medicine, Motol University Hospital, Prague, Czechia; ^9^Institute of Neuropsychiatric Care, Prague, Czechia; ^10^Psychiatry, Hospital District of Helsinki and Uusimaa, Helsinki, Finland; ^11^Adult Partial Hospitalization Program, Department of Psychiatry, Universitary Hospital Louis Mourier, Colombes, France; ^12^Health Service Executive, Dublin, Ireland; ^13^Vilnius City Mental Health Center, Vilnius, Lithuania; ^14^Lentis, Zuidlaren, Netherlands; ^15^European Psychiatric Association—Early Career Psychiatrists Committee, Wroclaw, Poland; ^16^County Emergency Clinical Hospital, Pius Brînzeu, Liaison Psychiatry, Timisoara, Romania; ^17^Department of Psychiatry, Faculty of Medical Sciences, University of Kragujevac, Kragujevac, Serbia; ^18^Psychiatric Inpatient Unit, Department of Psychiatry, Hospital Universitario Nuestra Señora de Valme, Seville, Spain; ^19^Department of Advanced Biomedical Sciences, Federico II University of Naples, Naples, Italy; ^20^School of Clinical Medicine, Shanghai University of Medicine and Health Sciences, Shanghai, China; ^21^University of Health Sciences, Erenköy Mental Health and Neurological Diseases Training and Research Hospital, Istanbul, Turkey; ^22^National Centre for Mental Health, MRC Centre for Neuropsychiatric Genetics and Genomics, Cardiff University, Cardiff, United Kingdom; ^23^Section of Women's Mental Health, King's College, Institute of Psychiatry, Psychology and Neuroscience, London, United Kingdom; ^24^University Hospital Center “Mother Theresa”, Tirana, Albania; ^25^Clinic of Psychiatry, University Clinical Center of the Republic of Srpska, Banjaluka, Bosnia and Herzegovina; ^26^Department of Psychiatry in Medical University, University Hospital “Aleksandrovska”, Sofia, Bulgaria; ^27^Unit for Social and Community Psychiatry, World Health Organization (WHO) Collaborating Centre for Mental Health Services Development, Queen Mary University of London, London, United Kingdom; ^28^Institute of Biomedical Sciences Abel Salazar, University of Porto, Porto, Portugal; ^29^Hospital de Magalhães Lemos, Porto, Portugal

**Keywords:** addiction psychiatry, addiction, EFPT, psychiatry trainees, psychiatry training

## Abstract

**Background:** Although psychoactive substance use disorders (PSUDs) are a domain of mental health, addiction psychiatry is only formally recognized as a subspecialty in a few European countries, and there is no standardized training curriculum.

**Methods:** A 76-item questionnaire was developed and disseminated through an online anonymous data-collecting system and hand-to-hand amongst psychiatric trainees from the 47 European countries of the Council of Europe plus Israel and Belarus.

**Results:** 1,049/1,118 psychiatric trainees from 30 European countries completed the questionnaire. Fifty-nine-point nine percent of trainees stated to have training in addictions. Amongst the trainees who described having training in addictions, 43% documented a not well-structured training and 37% an unsatisfactory training, mainly due to poor acquired knowledge. Overall, 97% of trainees stated that addiction represents a core curriculum for their training. Overall, general adult psychiatric trainees reported a better knowledge in addictions, compared to trainees in child and adolescent psychiatry.

**Conclusion:** Despite a growing spread of PSUDs in European countries, addiction psychiatry is a relatively poorly trained field within psychiatry training programs. Further research should investigate reasons for poor training and timings of the educational activities to optimize experiential education training in addiction psychiatry.

## Introduction

According to the Global Burden Disease study (1), alcohol, tobacco, and illicit substance use significantly determine the global burden of disability, morbidity and mortality, being considered amongst the top four health burdens across many upper-middle and high-income countries. Mental and behavioral disorders due to psychoactive substance use include different conditions caused by the intake of medically or not medically prescribed psychoactive substances (2). Psychoactive substance use disorder (PSUD) was firstly coded as a discrete diagnostic category both in the American Psychiatric Association (APA) Diagnostic and Statistical Manual (DSM)-3rd edition (DSM-III) and in the World Health Organization (WHO) International Classification of Diseases and Related Health Problems (ICD)-9th edition of the (2–4). The current Diagnostic and Statistical Manual-5th edition (DSM-5) (5) amalgamated the abuse and dependence under a single category named “Substance Use Disorder” whilst the ICD-11 beta draft (6) described substance dependence (not substance use) (ICD: F10.xx to F19.xx) as a “*disorder of regulation of the use of a psychoactive substance arising from repeated or continuous use of the substance* […]” (5, 6). Overall, PSUD may largely differ in severity and intensity in their psychopathological and clinical manifestation, i.e., ranging from an uncomplicated intoxication to the development of clinically significant psychotic disorders or other psychopathological and/or clinical manifestations) (2).

People with PSUD, including those classified as affected with a dual disorder, have been considered, compared to the general population, at higher risk of developing a range of medical and psychiatric disorders in comorbidity (7–9). Overall, PSUD subjects, particularly those with concurrent mental disorders, are overall associated with poorest outcomes, higher psychopathological severity and an increased rate of risky behaviors (i.e., hypersexuality, syringes/needles sharing, etc.) which can predispose them to an increased occurrence of serious infection diseases like Human Immunodeficiency Virus/Acquired Immune Deficiency Syndrome (HIV/AIDS) and Hepatitis C Virus (HCV), compared to the general population (10). Moreover, people with PSUD display a worsen psychosocial impairment (e.g., unemployment and homelessness) and they can more likely be involved in criminal and antisocial behaviors, compared to people affected by other mental disorders with a concurrent substance and/or alcohol use disorder (8, 11, 12).

However, although the PSUDs are fully considered among the mental and behavioral disorders, the contribution of psychiatrists, early career psychiatrists (ECPs) and psychiatry trainees into this clinical and research field, should be better developed. For instance, addiction psychiatry (sometimes named as addiction medicine) appears not to be adequately and homogeneously incorporated within the psychiatric training, across all European countries. Furthermore, psychiatry trainees' levels of knowledge and experiences in addiction psychiatry may greatly vary across European countries and cultures. As already documented by the 2014 WHO Global Survey on Resources for Prevention and Treatment of Substance Use Disorders, around 37% of the 155 responding countries do not provide adequate access to the post-graduate training programme for professionals working in PSUD treatment (13). Globally almost 30% of countries did not report a dedicated training programme for the treatment and the management of PSUD patients (52% of low-income countries vs. 16% of high-income countries), being mainly included in a short cycle tertiary education programme (48%). Overall, 95% of countries documented that psychiatrists are commonly involved in the treatment of people with PSUD, followed by psychologists, who are involved in PSUD treatment and management in around 86% of the countries. Furthermore, more than 80% of European countries reported the availability of a post-graduate training programme for the treatment and management of PSUD for psychiatrists (14).

Contextually, psychiatrists and psychiatry trainees' attitudes toward PSUD patients largely differ across different countries and cultures, where people with PSUD are generally more exposed to psychiatrists' and health professionals' negative attitudes/perception as well stigmatizing behaviors, and language (15). Stigmatizing behaviors and attitudes displayed by both psychiatrists and other physicians may lead to an inadequate and inhomogeneous physical, mental health care and treatment, including prescribing non evidence-based pharmacological/not pharmacological treatments, prescribing an inadequate/insufficient posology and duration of therapy. Moreover, use of potentially stigmatizing language may lead mental health professionals to a poor/inadequate communication with their PSUD patients, displaying an overall judgmental and unempathetic attitude, and other problematic and potentially stigmatizing behaviors (16–19).

The present study aimed at evaluating the organization of the addiction psychiatry training, trainees' satisfaction, trainees' attitudes toward people who use psychoactive substances and addiction psychiatry, and how psychiatric trainees manage psychopharmacology and pharmacotherapy in the most common clinical presentations of people with PSUD and their levels of confidence/perceived competence in the field of addiction psychiatry.

## Methods

### Study Design

The EFPT-PSUD Study has been an international cross-sectional survey of European psychiatry trainees carried out in the context of the European Federation of Psychiatric Trainees (EFPT), the umbrella organization of the national trainees' associations in psychiatry in Europe (20, 21). Among the framework of the EFPT, a working group specifically dedicated to the PSUD developed a self-administered survey that was disseminated at European level, by involving both Child and Adolescent Psychiatry (CAP) and General Adult Psychiatric (GAP) trainees.

#### Pilot Phase

All active members of the EFPT-PSUD Working Group, constituted during the 2014 EFPT Forum in London (22) and initially comprising national representatives from 5 countries (Italy, Croatia, Lithuania, Denmark, and Estonia), firstly conducted a preliminary overview about the current state-of-the-art regarding the training in addiction psychiatry in the European CAP/GAP training programs, and subsequently developed the survey. The survey was initially piloted amongst the members of the EFPT-PSUD Working Group.

#### Full Study Phase

The previously developed survey was circulated at the European level both to CAP and GAP trainees. The survey was circulated to the national representatives of each 47 European countries of the Council of Europe plus Israel and Belarus.

The European countries not represented in the survey were those not able to identify a National Coordinator who would take over the responsibility of the study or those unable to collect at least 10 completed questionnaires from their own country.

### Instrument

The questionnaire was a 76-item self-report survey (Appendix 1 in the Supplementary Material). The questionnaire consisted of: (a) single answer and/or multiple answer questions (i.e., for evaluating trainees' knowledge in a specific field); (b) an increasing five-item Likert scale (i.e., for evaluating attitudes and interests toward the addiction medicine and psychiatry); and, (c) a series of open-ended questions (i.e., asking for further specification and/or clarification of the provided answers). In particular, the section on general knowledge on addiction consists of 36 items in which each question correctly answered gave 1 point (range score: 0–36). This section was developed by GDP, following the evidence-based practices of the Substance Abuse and Mental Health Services Administration (SAMHSA) (https://www.samhsa.gov/ebp-resource-center).

For the present article, we have focused on the following sections of the survey:

- General socio-demographic section;
- General information about training in GAP (General Adult Psychiatry) or Child and Adolescent Psychiatry (CAP), including experiences (if any) on addiction psychiatry;
- General attitudes and interest toward addictions, addiction psychiatry;
- Level of knowledge about addictions, addictive disorders, including treatment.

### Data Collection

One national coordinator per each of 47 European countries of the Council of Europe plus Israel and Belarus facilitated the delivery of the survey through an online data collecting system (https://www.surveymonkey.com/r/EFPT-PSUDstudy) and/or, if necessary, delivering the questionnaire hand-by-hand, in a paper form (Appendix 1). The questionnaire was circulated in English across all European countries (in French language in France) and no translation in other languages was deemed necessary, as psychiatric trainees were deemed by their national coordinators to have sufficient command of English to reliably answer the questions (i.e., this was preliminarily evaluated by each national coordinator). Data were collected from 15th August 2015 to 15th October 2016. Annual EFPT forum as well as European and national congresses or educational events were chosen to reach out to all CAP/GAP trainees in each country or to involve national coordinators, needed for those countries still not represented in the sample of the survey. Moreover, European contact e-mail databases were periodically used to disseminate the link for the online survey (https://www.surveymonkey.com/r/EFPT-PSUDstudy). All hand-to-hand questionnaires completed were subsequently entered into the online study database by the National Coordinator via the online survey tool Survey Monkey. The online survey link was only accessible by invitation.

#### Inclusion Criteria

The inclusion criteria were: (i) being a CAP/GAP trainee, defined as a fully qualified medical doctor enrolled in a nationally recognized specialist training programme in CAP or GAP; (ii) belonging to one of the 47 European countries of the Council of Europe plus Israel and Belarus.

The participant countries included in the present analysis were those countries of whom each CAP/GAP National Coordinator was able to collect at least 10 completed questionnaires [not considering the last section regarding Novel Psychoactive Substances (NPS)]. Those countries with a National Coordinator who took responsibility to take part in the study but did not reach an enough minimum number of completed questionnaires were excluded in the present analysis (Greece, Belgium, Germany, Slovakia, Ukraine, Sweden, Denmark, and Israel). Amongst these, the following European countries participated in the present survey with a valid number of filled questionnaires: Albania, Austria, Azerbaijan, Belarus, Bosnia and Herzegovina, Bulgaria, Croatia, Czech Republic, Estonia, Finland, France, Hungary, Ireland, Italy, Kosovo, Latvia, Lithuania, Moldova, Netherlands, Norway, Poland, Portugal, Romania, Russia, Serbia, Slovenia, Spain, Switzerland, Turkey, and UK.

#### Ethics Approval and Consent

The survey was conducted according to the principles of good scientific practice, which was supported by previous EFPT-sponsored psychiatry trainees' surveys (23). Ethical approval for the study has been sought and granted by the School of Pharmacy Ethics Committee at the University of Hertfordshire (December 15, 2010, reference code PHAEC/10-42), with a further extension of the approval granted in November 2013. The patients/participants provided their written online informed consent to participate in this study.

Before filling out the survey which was self-administered anonymously, all participants were asked to give written online informed consent before, as legally and ethically required.

### Statistical Analysis

Data was analyzed using the Software Package for Social Sciences for Windows v. 24.0 (SPSS 24) (IBM Corp, Armonk NY). Categorical variables were summarized as *n* (%), and continuous variables as means [standard deviation (SD)]. Pearson's χ^2^-tests were used to compare demographic and categorical variables, such as the trainees' attitudes toward addiction psychiatry. Student's *t*-tests and one-way analysis of variance (ANOVA)-tests were used to compare continuous variables, including comparisons of training experiences. Ordinal regression was used to model the predictors of trainee satisfaction. Variables added to the model included trainees' sub-specialty and percentage of training completed. The significance level was set *a priori* at *p* ≤ 0.05, and all hypotheses were two-tailed.

## Results

### Sampling and Sample Characteristics

**Figure 1:**
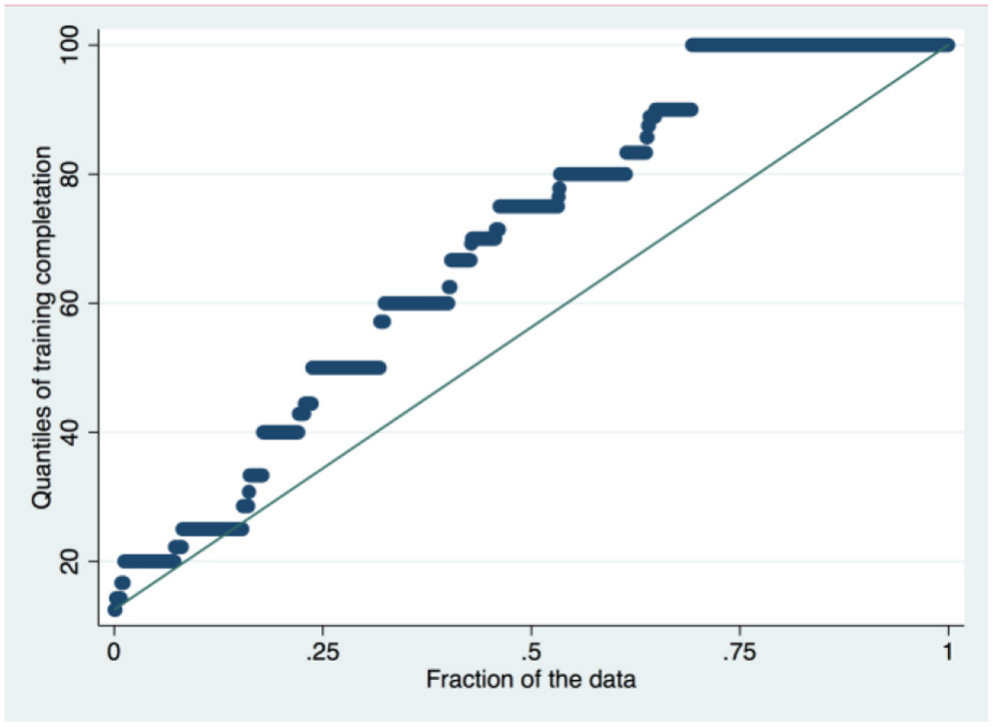
Distribution of training completion.

The total number of questionnaires correctly filled during the collection process and afterwards included in the analysis was of 1,118, amongst all trainees in Europe who took part in the survey. However, after excluding missing data (i.e., including only complete questionnaires) only 1,049 responses were included (Table 1). There were differences in the gender distribution, being most of them women (68.7%) and this difference is statistically significant when we stratified the sample by subspecialty (χ^2^ = 25, *p* < 0.001), being 84.6% of the CAP sample represented by women, whilst in the GAP sample, a percentage of 64% was represented by women, by reaching a total amount of GAP and CAP trainees of 936 (after excluding those trainees in forensic psychiatry or others with an unspecified other psychiatry training). The mean age of respondents was 30.48 (±4.84) years, without any statistically significant differences between GAP and CAP samples. The majority (73.8%) were GAP trainees, whereas 15.4% were CAP trainees, whilst around 10.6% of the sample did not specify if they are GAP or CAP trainees. Amongst the respondents, the total number of years required to complete GAP and CAP training programs may largely differ across European countries. To adjust the analysis for this confounder, it was calculated the percentage of progression/completeness of individual training for each country, in order to measure the most reliable and objective variable. This variable reported that in an average of 67.4% of the total sample, CAP/GAP trainees were in the last quantile of their training programme, without any statistically significant difference between GAP and CAP (see Figure 1). The CAP/GAP trainees overall belong to 30 different countries, with the highest proportion of respondents amongst those training in France (16.3%), followed by Italy (5.7%), Spain (5.1%) and the UK (5.0%). See Table 1 for further demographic features.

**Table 1:** Demographic characteristics.

	**Total**	**GAP (*N* = 774)**	**CAP (*N* = 162)**	**Significance**
	**Mean (SD)**	**Mean (SD)**	**Mean (SD)**	***t***
**Age**	30.48 (4.84)	30.43 (4.58)	30.79 (5.24)	*p* = 0.899
**Training completed (%)**	67.37 (28.01)	67.77 (28.44)	66.95 (28.64)	*p* = 0.716
	**Frequency (%)**	**Frequency (%)**	**Frequency (%)**	***χ^2^***
**Gender**				*p* < 0.001
Male	328 (31.3%)	269 (34.8%)	25 (15.4%)	
Female	721 (68.7%)	505 (65.2%)	137 (84.6%)	
**Country of training**				*p* = 0.221
Croatia	38 (3.6%)	26 (3.4%)	5 (3.1%)	
Czech Republic	36 (3.4%)	28 (3.65%)	8 (4.9%)	
Finland	44 (4.2%)	32 (4.1%)	8 (4.9%)	
France	171 (16.3%)	101 (13.0%)	30 (18.5%)	
Ireland	40 (3.8%)	20 (2.6%)	5 (3.1%)	
Italy	57 (5.4%)	50 (6.5%)	7 (4.3%)	
Lithuania	45 (4.3%)	35 (4.5%)	10 (6.2%)	
Netherlands	35 (3.3%)	23 (3.0%)	3 (1.9%)	
Poland	47 (4.5%)	38 (4.9%)	6 (3.7%)	
Portugal	42 (4.0%)	37 (4.8%)	5 (3.1%)	
Romania	45 (4.3%)	39 (5.0%)	6 (3.7%)	
Slovenia	33 (3.1%)	22 (2.8%)	9 (5.6%)	
Spain	53 (5.1%)	47 (6.1%)	1 (0.6%)	
Switzerland	34 (3.2%)	27 (3.5%)	7 (4.3%)	
Turkey	40 (3.8%)	31 (4.0%)	9 (5.6%)	
UK	52 (5.0%)	30 (3.9%)	5 (3.1%)	
Other	237 (22.6%)	188 (24.3%)	38 (23.5%)	

*GAP, General Adult Psychiatry; CAP, Child and Adolescent Psychiatry; SD, Standard Deviation; UK, United Kingdom*.

### Trainees' Experience, Satisfaction, and Training in Addiction Psychiatry

Amongst those who answered the question “*Have you performed part of your psychiatric training in the treatment of patient with substance use disorder?*”, only 59.9% of trainees reported to have spent part of their training in addiction psychiatry settings, with a statistically significant difference between GAP and CAP trainees (*p* = 0.018). Amongst those trainees who declared to have received training in addiction psychiatry during their psychiatry training, only 43% described that the PSUD training was not well-structured due to several reasons. First, the addiction training program is often too short to allow trainees to deepen knowledge on all theoretical and practical aspects of addiction psychiatry; second, during the addiction training program, CAP/GAP trainees are often alone in the management of PSUD patients (often without a dedicated supervisor/mentor); third, the addiction training program usually consists in a mere observership experience (without a practical frontline experience). Amongst those trainees who had training in addictions only 37% of them declared that they were not satisfied about the level of training offered, mainly stating lack of enough acquired skills and knowledge in the field, largely below their initial expectations. There was no significant difference in the percentage of training completed amongst those trainees who reported being satisfied with their addiction psychiatry training, compared to those trainees who did not document an enough level of satisfaction [F_(1,555)_ = 2.244, *p* = 0.135]. Trainees with larger caseloads had generally progressed further in their training, compared to those trainees with smaller caseloads [F_(5,551)_ = 6.487, *p* < 0.001]. Most of the sample (97%) agreed or strongly agreed that addiction represents a core curriculum for training. Subspecialty was a significant predictor of satisfaction with training (β = 1.713; *p* = 0.042), being GAP trainees overall more satisfied, compared to CAP trainees, even though this finding is not strictly correlated by the percentage of training completed (β = 1.005; *p* = 0.176).

### Trainees' Attitudes Towards People Who Use Psychoactive Substances and Addiction Psychiatry

Approximately one third of the sample (33.27%) agreed or strongly agreed to be confident with their basic skills needed/requested necessary to work in addiction settings after their training (χ^2^ = 82.864; *p* < 0.001). Interestingly, on the other hand, around 66.9% of the trainees agreed or strongly agreed that “*Addiction psychiatrists are usually less skilled than their colleagues working in GAP/CAP*” (Table 2). Moreover, around 75.7% disagreed or strongly disagreed that addictions are mental disorders; similarly, 77.8% of the sample agreed or strongly agreed that people with drug addiction cannot be recovered (Table 2).

**Table 2:** Attitude of trainees who have/haven't performed part of their training in the treatment of a patient with addiction.

		**Have you performed part of your psychiatric training in the treatment of patients with addiction?**		
		**Yes**	**No**	
Illicit drugs (e.g., heroin) addicted are good people	Strongly agree	9	3	*χ^2^* = 8.773 *p* = 0.067
	Agree	33	22	
	Neither agree or disagree	303	236	
	Disagree	101	52	
	Strongly disagree	62	60	
I don't feel confident with my skills to work in addiction	Strongly agree	36	22	*χ^2^* = 82.864 *p* < 0.001
	Agree	178	44	
	Neither agree or disagree	125	84	
	Disagree	146	181	
	Strongly disagree	23	42	
I think that people with drug addiction cannot recover	Strongly agree	138	88	*χ^2^* = 3.872 *p* = 0.424
	Agree	257	188	
	Neither agree or disagree	87	75	
	Disagree	25	19	
	Strongly disagree	1	3	
Addiction is a mental disorder	Strongly agree	5	4	*χ^2^* = 6.263 *p* = 0.180
	Agree	25	15	
	Neither agree or disagree	59	61	
	Disagree	262	198	
	Strongly disagree	15	95	
Addiction psychiatrists are usually less skilled than their colleagues working in general adult and child adolescent psychiatry	Strongly agree	159	130	*χ^2^* = 6.565 *p* = 0.161
	Agree	181	140	
	Neither agree or disagree	104	73	
	Disagree	57	24	
	Strongly disagree	7	6	

**Figure 2:**
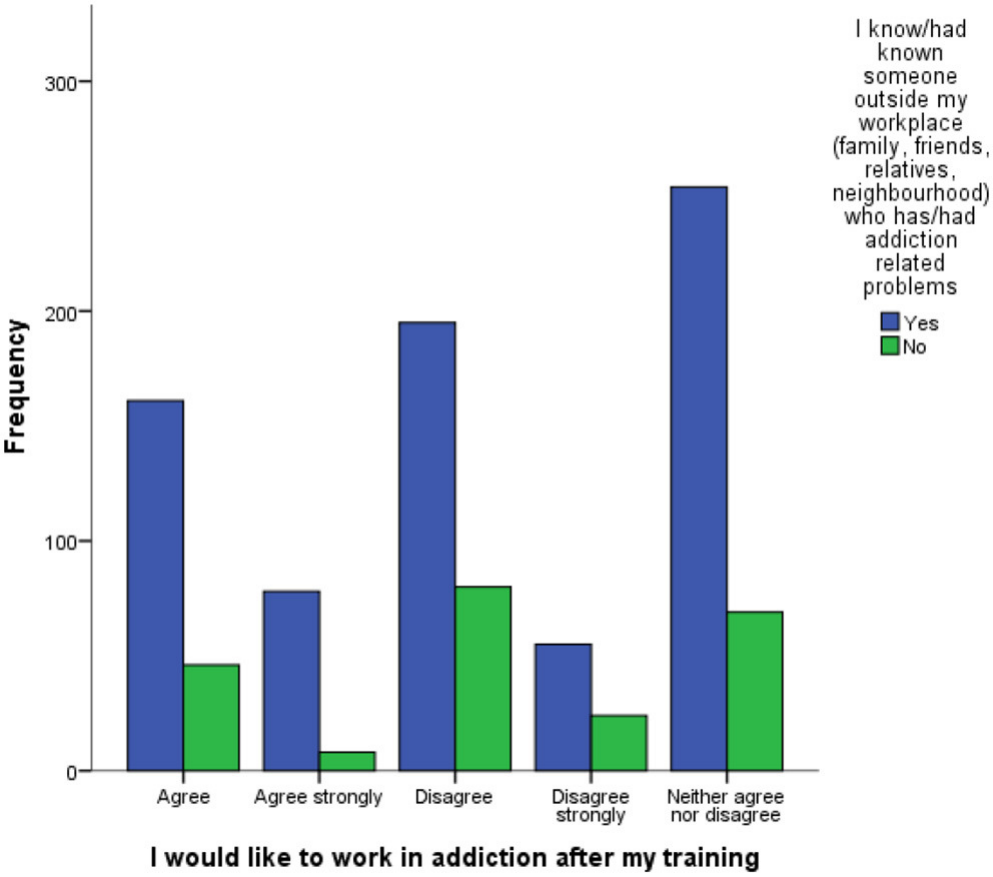
Frequency of trainees who would like to work in addiction following completion (by those who have/haven't known someone outside the workplace who has had an addiction related problem).

Over three-quarters of respondents (76.1%) knew/had previously known someone outside of their workplace with an addiction-related problem (Table 3). The findings showed that those who knew/had known someone with addiction related problems were significantly associated with a stronger desire to work in the addictions after their training [χ^2^(4) = 16.311, *p* = 0.003] (Figure 2).

**Table 3:** Attitude of trainees who have/haven't known someone outside their workplace with addiction related problems.

		**I know/had known someone outside my workplace (family, friends, relatives, neighborhood) who has/had addiction related problems**		
		**Yes**	**No**	
I am afraid to work with persons with cocaine addiction	Strongly agree	204	39	*χ^2^* = 14.623 *p* = 0.006
	Agree	309	101	
	Neither agree or disagree	92	42	
	Disagree	62	20	
	Strongly disagree	7	5	
I am afraid to work with persons with alcohol	Strongly agree	283	68	*χ^2^* = 8.305 *p* = 0.081
	Agree	298	97	
	Neither agree or disagree	64	30	
	Disagree	25	11	
	Strongly disagree	4	1	
Addiction is a mental disorder	Strongly agree	8	1	*χ^2^* = 14.525 *p* = 0.006
	Agree	34	6	
	Neither agree or disagree	83	37	
	Disagree	339	121	
	Strongly disagree	210	42	
Individual psychotherapy should be preferred in treating addiction	Strongly agree	14	1	*χ^2^* = 12.680 *p* = 0.013
	Agree	125	45	
	Neither agree or disagree	236	93	
	Disagree	238	52	
	Strongly disagree	61	16	

### Trainees' Basic Knowledge and Confidence/Perceived Competence in Addiction Psychiatry

**Figure 3:**
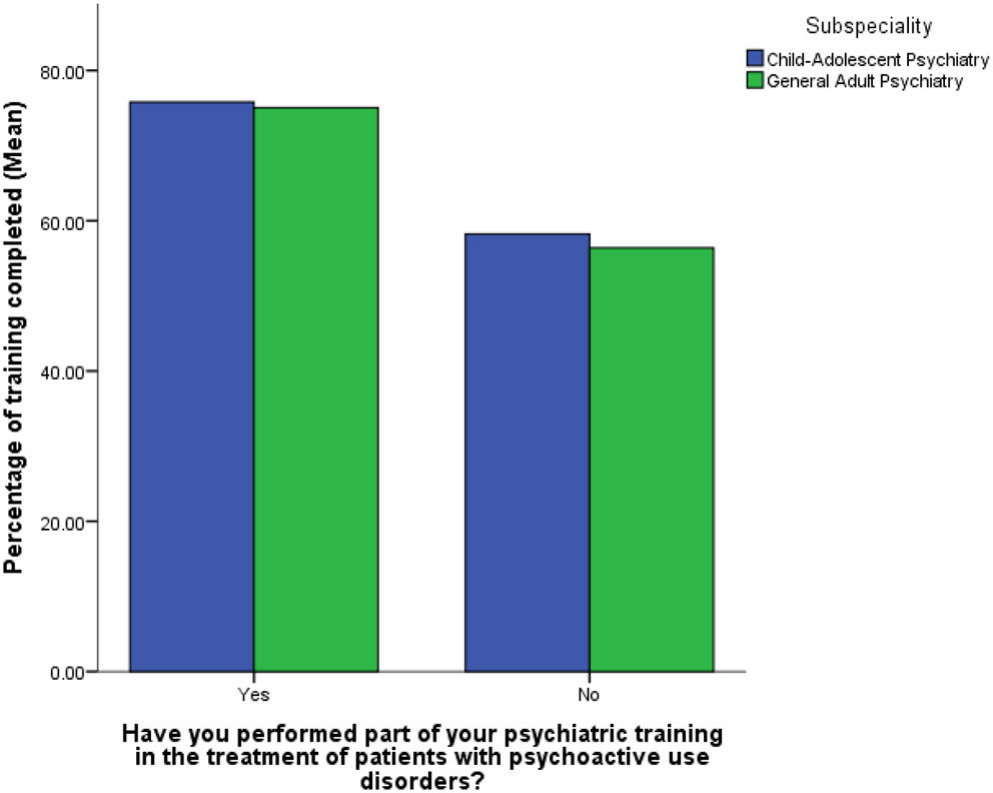
Mean percentage of training completed in those who have/haven't worked with patients with psychoactive use disorders during their training (by sub-specialty).

**Figure 4:**
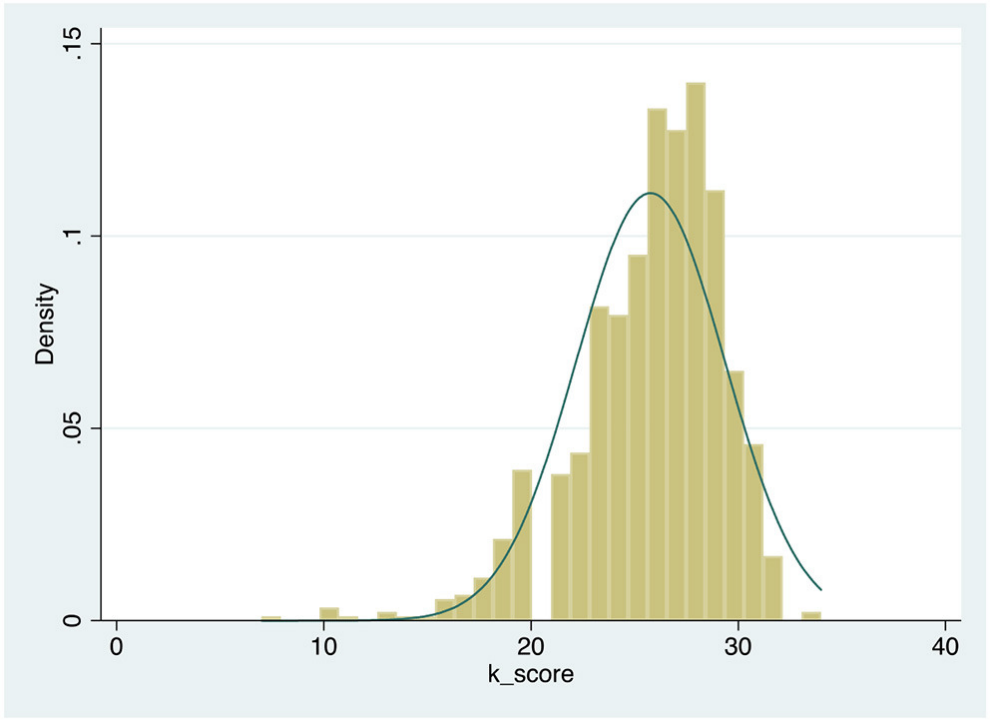
Distribution of the knowledge score.

Respondents who had treated someone with an addiction-related condition significantly declared to have almost completed their training, compared to those trainees who had not [F_(1,991)_ = 99.155, *p* < 0.001] (Figure 3). Figure 4 represents the graphical distribution of the knowledge score, by indicating that most trainees responded correctly to most of the questions regarding their general and specific knowledge of addiction psychiatry (mean average 25.77 ± SD 3.59), with a minimum score of 7 and a maximum score of 34 (skewness = −0.956). There was no significant difference in terms of the most prevalent addiction-related condition that was treated/observed during their addiction psychiatry training [F_(4,479)_ = 1.523, *p* = 0.194]. However, those trainees who had treated alcohol withdrawal syndrome, delirium tremens, opioid withdrawal syndrome, or substance induced-psychosis were significantly more senior in their level of training completeness, compared to those trainees who had not treated these addiction-related conditions who were more junior (all *p*-values < 0.001). Similarly, those trainees prescribing acamprosate, naltrexone, methadone, and buprenorphine were also significantly further in their training than those who did not prescribe a medication for an addiction (all *p*-values < 0.001). In addition, GAP trainees more likely reported to have treated a person affected with an addiction during their training, compared to CAP trainees [χ^2^_(1)_ = 8.328, *p* = 0.004]. Likewise, GAP trainees more likely reported to have prescribed medication for an addiction-related condition, compared to CAP trainees [χ^2^_(1)_ = 9.482, *p* = 0.002]. Furthermore, GAP trainees reached higher scores, compared to those undergoing CAP training, when questioned about their general and specific knowledge of addictions [F_(1,802)_ = 14.181, *p* < 0.001]. Moreover, GAP trainees were more likely aware of the existance of legal highs/smart drugs/novel substances, compared to CAP trainees [χ^2^_(2)_ = 25.663, *p* < 0.001]. However, when the knowledge score includes in the analysis also those questions about legal highs/smart drugs/novel substances, there was no significant difference in the total score between GAP and CAP trainees [F_(1,531)_ = 0.524, *p* = 0.470].

## Discussion

### Key Findings and Comparison With the Literature

PSUD have been historically perceived as personal, family, social, moral, or criminal issues rather than a health condition (24). Therefore, subjects with PSUD have been supposed to be better managed at the individual, family or justice level (i.e., through existing social infrastructure or civil and criminal justice interventions) (24). Indeed, criminalization of people with PSUD exacerbated their perceived and experienced stigma, avoidant attitudes and behaviors of contempt, by worsening their marginalization and poor access to adequate treatment and care (24). People with PSUD tend to be stigmatized due to their use of drugs and drug-seeking behaviors (24). Moreover, other PSUD-related risky behaviors, such as speeding/dangerous driving, violence, aggressiveness, and impulse dysregulation, are barely seen as part of a complex disorder, so that people with PSUD are usually rejected by the society due to the supposed moral valence of these behaviors (24, 25). These patients may also be seen as a burden for the healthcare system, by indeed increasing the disparities of cares, the risk to not adequately provide evidence-based and effective treatments (19, 25). Due to this disadvantageous framework, patients with PSUD may develop a self-stigmatizing attitude as well (e.g., a subjective process characterized by negative feelings about own self, maladaptive behaviors, stereotype endorsement resulting from individual's experiences/perceptions/feelings and anticipation of negative social reactions) (26–29). In fact, potentially “stigmatized” attitudes and behaviors, overly provided by healthcare professionals, including psychiatrists and psychiatry trainees, may be potentially trigger and maintain these self-stigmatizing attitudes, as already reported in the literature and confirmed by our findings (16, 19, 26–29). Furthermore, subjects with PSUD are symbolically associated with a range of other stigmatized health conditions, including HIV/AIDS, HCV, risk and disinhibiting behaviors such as impaired driving, prodigality, criminality, risky sexual behaviors, and social issues (30, 31). Stigmatizing beliefs and behaviors about PSUD may be influenced by the level of knowledge (and education) about these mental health conditions and the personal experience with people affected with PSUD. Furthermore, it has been reported that media portrayal of people with PSUD and media coverage/level of news disseminated about significant and impactful related events, mainly occurring due to a drug intoxication and/or drug dependence/abuse/misuse, can significantly increase these stigmatizing beliefs and attitudes (29, 32).

Furthermore, addictions have not been historically recognized as conditions requiring a medical, psychological and psychopharmacological treatment (19). This is in line with our findings in which most GAP and CAP trainees declared that the addictions are not mental disorders. In fact, as previously documented in the literature, this overall consideration regrading PSUD appears to be widely spread not only at the general population level but also amongst mental health professionals who overall reported negative and pessimistic views about PSUD, people with PSUD and do not routinely screen patients in daily practice for addictions (15, 26, 33, 34).

However, the individual perceptions and attitudes towards people with PSUD may largely vary according to different factors. For instance, people are less likely to endorse the stereotype of violence together with a negative connotation of addiction disorders, if they have had direct contact with people (or also family members or close friends) who were affected with PSUD or did not experience violent acts by people affected with PSUD (35). This is comparable with our findings which demonstrated that those trainees who have/had experience with people with a PSUD significantly declared to have a stronger desire to work in the addiction field and with subjects with PSUD after their training.

Furthermore, despite a compelling need for PSUD treatment in Europe, mental healthcare professionals (including psychiatry trainees) overall appear poorly or neither trained, nor especially eager to accept/tolerate patients with PSUD (15, 33, 34, 36). In general, psychiatrists do not feel competent/confident in treating addiction disorders, do not like working with patients affected with PSUD and do not find rewarding treating patients with PSUD (33, 37, 38). A lack of (practical) experience and/or an inadequate (theoretical and practical) training in addiction psychiatry may indeed result in an endless loop of incompetence and neglect regarding the addiction psychiatry, amongst mental health care professionals. However, despite the evidence demonstrating the need to improve addiction medicine's training not only amongst psychiatry trainees but also amongst all physician trainees, most medical students and CAP/GAP trainees generally receive an inadequate (practical and theoretical) training in the field of addiction medicine/psychiatry (33, 39, 40). Moreover, most CAP/GAP trainees generally display lacking core clinical and therapeutic competences, as required for working with patients with PSUD (33, 39, 40). Although formal addiction training within the medical field has been closely tied to psychiatry, psychiatric training generally provides a poor improvement and a limited level of knowledge over medical school, about addictions (39, 40). These considerations are particularly significant in the European countries, whereas there are several inequalities and heterogeneous training levels in addiction psychiatry, as documented by our findings. Furthermore, most CAP and GAP trainees reported to be less skilled in the addiction field, compared to other fields of psychiatry. Interestingly, there are not statistically significant differences between GAP and CAP trainees regarding this finding. This appears particularly relevant if we consider that CAP trainees should possess a comprehensive experience including behavioral, psychosocial and addiction problems particularly amongst youngsters/adolescents who have been well-demonstrated to be those patients more frequently exposed to drugs and/or other addictive behaviors, but also those patients more vulnerable toward the new onset of mental disorders associated with a PSUD (41).

Furthermore, an insufficient training experience with patients with PSUD, along with the lack of a highly-specialized faculty (i.e., short addiction training experience, lack of a supervisor/mentor during the addiction training, and poor quality of addiction training), may overall lead to a discouraging training experience amongst CAP/GAP trainees, as reported in our study. Overall, one could argue that this general psychiatry trainees' attitudes and perceptions towards the addiction psychiatry might discourage trainees' interest and willingness to deepen the management and therapy of patients with PSUD, independently by their level of psychiatry training, as well as their interests in working in addiction psychiatry (38). Renner et al. (38) described the following main predictors of poor perception of careers in addiction medicine by GAP trainees: (a) the poor/not enough/lacking experience with patients with PSUD; (b) the perceived sensation and feeling to work with “difficult” patients; (c) the lack of a competent training in the addiction; (d) an overemphasis, during psychiatry training, about the detoxification process rather than a long-term rehabilitative and care program for the addiction-related conditions. Miller et al. (33) identified the following hypothesized barriers/determinants explaining the different attitudes and practices of medical students, trainees and physicians towards addiction psychiatry: (a) lack of acceptance of a medical model for addictive disorders; (b) lack of positive and/or optimistic attitudes about patients with PSUD, by accepting the prevalent stereotype of subjects with PSUD as those patients whose social and medical prognosis is poor; (c) curricula deficits throughout the Continuum Medical Education (CME) in the field of the addiction psychiatry/medicine, particularly the total time devoted to addictive disorders during the medical school and psychiatry training; (d) lack of parity and physician advocacy in medical education; (e) prejudices and misunderstandings about addictive disorders, along with ungrounded fears of huge costs connected with addiction treatment and the perception that addiction treatment owns a low ratio of benefits to costs; (f) personal and/or family history of drug and/or alcohol disorders. Conversely, Rush et al. (42) found that the factors associated with more positive attitudes towards the treatment of addictive disorders and subjects with PSUD may be represented by: (a) the number of subjects with PSUD treated/visited; (b) the physicians' perceived effectiveness in the management of the addictive disorders; and, (c) the numbers of hours of CME specifically addressed on the addictive disorders.

However, as widely reported in the literature, the level of knowledge and education about PSUD and addiction psychiatry can positively influence mental health professionals' attitudes and interests towards the field of addictions, limit the misdiagnosis and potentially reduce improper and inadequate treatment regimens for these disorders (43–45), even though other studies demonstrated a deterioration in attitudes throughout medical school years and suggested a continued decline throughout the years of training, mainly due to time and resources spent for those subjects with PSUD (19, 46–49). The enhancement of these beliefs appears to be more significant when we compared those subjects with PSUD with those with AUD (49). As proposed by Miller et al. (33), to achieve an adequate level of education and training in addiction psychiatry, it should be ensured that all trainees reach an enough and adequate knowledge and skills in the diagnosis and treatment of the addictive disorders, by favoring the development of curricula for the addictive disorders in all medical schools, residency training programs and CME; by supporting the research and revising all discriminatory policies that create barriers to the implementation of curricula in addictive disorders; by providing the detection and intervention for students, trainees and physicians who have addictive disorders; and, by supporting the parity between the addictive disorders and other medical and psychiatric diseases.

### Main Strengths and Limitations

To the best of our knowledge, this has been the only study specifically investigating the levels of training, experiences, attitudes and perceptions as well as the level of perceived confidence and capacity in the management of people with a PSUD, carried out amongst European CAP/GAP trainees. The present survey also included a large sample size of CAP/GAP trainees in Europe (*n* = 1,118) which comprises many European countries (*n* = 30). Furthermore, collecting data from different European countries might lend strength to the generalization of these findings also to other WHO Regions, beyond European Region. Moreover, our study identifies gaps in knowledge by demonstrating that addiction psychiatry appears not to be adequately and homogeneously incorporated within the psychiatric training, across all European countries. Moreover, a key finding is represented by the significant number of recruited psychiatry's trainees who do not consider addiction as a psychiatric disorder.

Despite its original and poorly investigated topic, there are several limitations that should be here drawn up. Firstly, being a self-report questionnaire and partly online administered, potential recall, social desirability, and reporting biases may occur. Secondly, the sampling method may be hugely affected both by the fact that not in all European countries we reached an enough number of completed questionnaires or reached an available official national coordinator. In fact, some European countries initially included have been *a posteriori* excluded in our analysis as they did not reach an enough number of completed questionnaires (cut-off of 10 for each country), like Greece, Belgium, Germany, Slovakia, Ukraine, Sweden, Denmark, and Israel. Furthermore, sampling rates largely vary within different European countries, being some countries (i.e., Croatia, Finland, France, Ireland, Italy, Lithuania, Poland, Portugal, Romania, Slovenia, Spain, Turkey, and UK) most represented in our sample compared to Albania, Bosnia, Bulgaria, Czech Republic, Estonia, The Netherlands, Serbia, and Switzerland. The level of perceived confidence and knowledge in addiction psychiatry, being mainly based on a set of questionnaires, may also be susceptible to the updated information and new available and emerging pharmacological and not pharmacological treatments, may not completely reflect the current situation occurring at the time of writing of the present study. Moreover, the present study does not examine what happens once GAP/CAP residency is completed and the GAP/CAP enters career's practice. It should be relevant to document further data particularly regarding the level of attitude or perception of PSUD patients with added experiences and added continuing educational opportunities during their clinical career. Finally, the present study does not specifically define whether psychiatry trainees' attitudes differ towards caring for subjects with AUD and/or SUD.

### Relevance of the Findings and Implications for Practice, Policies, and Research

The present study provides significant and valuable information on the current European CAP/GAP trainees' level of experiences, training, perceived knowledge/competence, and subjective attitudes/perceptions towards the addiction psychiatry. These findings not only serve to investigate the current European situation in terms of level of subspecialty offered in the addiction psychiatry as well as the potential differences across all analyzed European countries, but they might also investigate those situations which should be implemented/enhanced as lacking in providing opportunities both in terms of internship (practical training) and knowledge (theoretical training) in the field of the addictions. Moreover, addressing the identified reasons/factors determining a different level of training in addiction psychiatry as well as a different level of interest CAP/GAP trainees, in strenghtening knowledge in this field might be a way to modulate and act on these factors, to improve the CAP/GAP training conditions in the field of addiction psychiatry (50). Regarding the need to improve all CAP/GAP training programmes, the standardization of curricula would be important to produce both GAP and CAP trainees able and capable (self-confident) in the management and correct identification of both physical and mental/behavioral PSUD-related conditions. This should be part of the essential core knowledge that should be indispensable for all psychiatric practice. In terms of the enhancement of GAP/CAP trainees' education/knowledge in the addiction medicine and psychiatry, an implementation of a mandatory addiction rotation during the CAP and GAP training program, could greatly improve the level of trainees' confidence and competence in identifying and dealing with all different addictive disorders. Furthermore, in CAP and GAP training, the need to develop and satisfy objective measurable educational criteria must be balanced with the acquisition of subjective skills needed to treat subjects with PSUD effectively (e.g., increasing empathy and not judgmental approach as well as addressing stigma), as well as reaching an enough comfort in working with PSUD patients and obtaining a minimum sense of mastery in the field of the addictions. Finally, it might be suggested to all European GAP and CAP training programmes to administer to all psychiatry trainees at the end of their training program, validated tools for assessing addiction psychiatry training and early identify potential deficits, such as the Addiction Training Scale (ATS) (51).

These findings may assist the decision-makers to implement strategies to adapt their national diversities in CAP/GAP training programmes and make them homogenous especially at the European level. The need for psychiatry trainees' education and experience in treating patients with addiction problems has been outlined. Lastly, although these preliminary findings may help in mapping the reality of this field of psychiatry, further studies are needed to focus on the main motivations underpinning the existing differences across European countries in terms of level of training in addiction psychiatry (i.e., cultural and/or religious factors, epidemiological motivations, etc.) and consequences of different experiences/training in the level of knowledge of a CAP/GAP trainees as well as their attitude/perception towards addictions in general and people who use psychoactive substances. Moreover, it would also be of interest to repeat the present survey with identical methodology every 4 or 5 years (being the average duration of CAP/GAP European training) to assess potential trends in these findings and attitudes/opinions of psychiatry trainees over time and evaluate if any enhancing intervention has been provided at European and national level concerning addiction psychiatry training and evaluate if any positive/neutral/negative impact was reached amongst psychiatry trainees' attitudes and knowledge.

## Conclusions

Despite the growing dissemination of addictive disorders across all European countries, addiction psychiatry seems to be an underdeveloped part of psychiatry within psychiatry training programmes. However, we found substantial consensus among all European psychiatry trainees that more education and experience in treating patients with addictive disorders should be guaranteed and be part of the core curricula in GAP and CAP training. Further research needs to be directed towards the causes of poor training as well as timings of these educational activities to optimize experiential education programs to be implemented within GAP and CAP training programs.

## Data Availability Statement

The raw data supporting the conclusions of this article will be made available by the authors, without undue reservation.

## Ethics Statement

The studies involving human participants were reviewed and approved by Ethical approval for the study has been sought and granted by the School of Pharmacy Ethics Committee at the University of Hertfordshire (December 15, 2010, Reference Code PHAEC/10-42), with a further extension of the approval Granted in November 2013. The patients/participants provided their written informed consent to participate in this study.

## Author Contributions

LO, IR, GDP, MPo, DQ, and MM conceived and conceptualized the study. GDP, LO, and IR performed the survey, the methodology and the ways to disseminate the survey across all European countries. IR and GDP mainly dealt with data curation, collection and analysis. DQ performed formal analysis of data collected reported in this article. A preliminary draft was written by GDP, LO, IR, and MPi. LO wrote, revised and edited the final draft. MPi supervised the work and contributed to the final editing of this manuscript. All other co-authors equally collected data from their respective countries and provided further final feedback to the draft.

## Conflict of Interest

The authors declare that the research was conducted in the absence of any commercial or financial relationships that could be construed as a potential conflict of interest.
