# Supplementary material for: Psychiatry Trainees' Attitudes, Knowledge, and Training in Addiction Psychiatry—A European Survey
Source: Front Psychiatry. 2021 Jan 8;11:585607. doi: 10.3389/fpsyt.2020.585607 (PMC7820719; doi:10.3389/fpsyt.2020.585607)
Supplement: Supplementary file 1 [file Data_Sheet_1.pdf]

ABOUT THIS SURVEY

What is it for?
The main aim of this survey is to find out how much psychiatric trainees and/or young psychiatrists know/have learned about addiction psychiatry and treatment in their training, their opinion and attitudes.
Who should fill it in?
You should fill in this questionnaire only if you are currently a **psychiatric trainee** *or* a **young psychiatrist within 5 years from your training completion**, and your training is/has been based
in Europe (Europe being the 47 countries of the Council of Europe plus Israel and Belarus) .
Who is running this survey?
This is a research project being conducted by the EFPT (European Federation of Psychiatric Trainees) working group on Psychoactive Substance Use Disorders (EFPT-PSUD WG; more info available at http://efpt.eu/wordpress/working-groups/psychoactive-substance-use-disorders-wg/).
How long does it take?
It takes around 15 minutes to complete it. We would really appreciate it if you could take the time to fill all five sections of the survey.
Your responses will be absolutely anonymous and confidential.
We do not need your name nor your contact details. To help protect your confidentially, the survey will not contain information that will personally identify you.
Your answers will be seen only by our research group. **The findings of this study will be used for research purposes only.**
If you have any questions about the research study, please contact: psud-wg@efpt.eu.

If you agree to fill in the questionnaire, first read these important notes:

The survey questionnaire has 5 sections:

A. General section about you;

B. General information on your training;

C. General attitudes and interest on addictions;

D. What you know about addiction;

E. Final section.

INFORMED CONSENT: **Please select your choice:** ☐ Agree ☐ Disagree

Ticking the "agree" box indicates that:
- You have read the above information
- You voluntarily agree to participate in this research
- You meet the above inclusion criteria
- Please, try to answer to all the questions
- If you are filling a PDF version, please save it after filling the survey

Thanks for your contribution.

A. GENERAL SECTION ABOUT YOU

A1. ***Age*** …………

A2. ***Gender*** ☐ Male ☐ Female

A3. ***Country of training*** ………………………………….

A5. ☐ ***Year of training*** : ….. / ….. (Year of training/Total years of training in your country) OR ☐ ***Specialist*** since year: …..

A6. ☐ ***General adult Psychiatry*** (GAP) or ☐ ***Child-Adolescent Psychiatry*** (CAP)

A7. I know/had known someone outside my workplace (family, friends, relatives, neighborhood) who has/had addiction related problems ☐ Yes ☐ No

B. GENERAL INFORMATION ON YOUR TRAINING

B1. ***Are psychiatrists involved in the field of addiction medicine, in your country ?***
☐ Yes ☐ No

B2. ***Does your psychiatric training give you the chance of training in addiction services within your country ?***
☐ Yes ☐ No

B3. ***During your training have you spent (or are you currently spending) a period of your traineeship in any addiction facility?***
☐ Yes ☐ No

B4. ***If B3=yes, how much is/was long this period?***
☐ < 3 months ☐ 3-6 months ☐ >6 months

B5. ***Does your psychiatric training include/included a mandatory experience in addiction services?***
☐ Yes ☐ No

B6. ***Does your training provides a sub-specialization in addiction psychiatry?***
☐ Yes ☐ No

B7. FACULTATIVE: ***Specify if your psychiatric training provides other possibilities/solutions (eg you have done such a training abroad).*** [open question]
………
………

If B3=yes, please fill in also the following questions (B8-B16):

B8. ***If you have already done or doing a training in addiction psychiatry (within your psychiatric training), Is/was the training well-structured?*** Yes ☐ No ☐

If not, explain why **[you can tick more answers]**:
- ☐ Too short period
- ☐ Too long period
- ☐ I am/was alone in managing patients
- ☐ It was just an observership
- ☐ other. Specify: ……………………………………………………………………………….

B9. ***If you have already spent/ are spending a training in addiction psychiatry (within your psychiatric training), Is/was the training satisfactory?***
☐ Yes ☐ No

If NO**, **explain why? **[you can tick more answers]**:
- ☐ Not interesting experience
- ☐ Not useful experience
- ☐ Do not/did not develop enough skills/knowledge
- ☐ The environment and patients were 'not for me'
- ☐ other. Specify: ……………………………………………………………………………….

B10. ***Which of the following drugs did you prescribe (even if under supervision) as pharmacological treatment of different addictions?* [you can tick more answers]**

☐ Acamprosate ☐ Naltrexone ☐ Methadone ☐ Buprenorphine ☐ Disulfiram ☐ None of them
☐ None of them

B11. ***Which of the following medical conditions did you manage (even if under supervision)?* [you can tick more answers]**

☐ Alcohol withdrawal syndrome ☐ Delirium tremens ☐ Opioid withdrawal syndrome ☐ Substance induced-psychosis

B15. ***The number of patients in your addiction training that I have approximately worked with is***:

☐ 0 ☐ <10 ☐ 10-30 ☐ 31-50 ☐ 51-100 ☐ >100

B.16 . ***Within the patients you worked with during your addiction training, most prevalent substance use disorders were* [you can tick one answer]**

☐ alcohol-related ☐ heroin/opiates-related ☐ cocaine/stimulants related
☐ cannabis-related ☐ smart drugs/legal highs-related

C. GENERAL ATTITUDES AND INTEREST ON ADDICTION PSYCHIATRY

**PLEASE TICK *ONE* BOX ON EACH LINE**	**Agree strongly**	**Agree**	**Neither agree nor disagree**	**Disagree**	**Disagree strongly**
C1. I agree that addiction is a relevant core curriculum for psychiatric training	☐	☐	☐	☐	☐
C2. I would like to work in addiction after my training	☐	☐	☐	☐	☐
C3. Illicit drugs (eg heroin) addicted are good people	☐	☐	☐	☐	☐
C4. I do not feel confident with my skills to work in addiction	☐	☐	☐	☐	☐
C5. I am at ease in working with persons with heroin addiction	☐	☐	☐	☐	☐
C6. I think that people with drug addiction cannot recover	☐	☐	☐	☐	☐
C7. Addiction is based on choice	☐	☐	☐	☐	☐
C8. Addiction is a mental disorder	☐	☐	☐	☐	☐
C9. I am afraid to work with persons with alcohol	☐	☐	☐	☐	☐
C10. Addiction is primarily a biological disease	☐	☐	☐	☐	☐
C11. Addiction is primarily influenced by society	☐	☐	☐	☐	☐
C12 I am afraid to work with persons with cocaine addiction	☐	☐	☐	☐	☐
C13. Licit drugs (eg alcohol) addicted are bad people	☐	☐	☐	☐	☐
C14. Individual psychotherapy should be preferred in treating addiction	☐	☐	☐	☐	☐
C15. Group psychotherapy should be preferred in treating patients with addiction	☐	☐	☐	☐	☐
C16. Clubs of treated patients, alcoholics/narcotics anonymous, and therapeutic communities should be considered in the recovery process from addiction	☐	☐	☐	☐	☐
C17. Addiction psychiatrists are usually less skilled then their collegues working in general adult and child adolescent psychiatry	☐	☐	☐	☐	☐

D. WHAT YOU KNOW ABOUT ADDICTION

PLEASE TICK *ONE* BOX ON EACH LINE

	Agree strongly	Agree	Neither agree nor disagree	Disagree	Disagree strongly
D1. Licit use of psychoactive substances (e.g. alcohol, tobacco) is less harmful for health than illicit use	☐	☐	☐	☐	☐
D2. Diseases related to psychoactive substance use are not reversible	☐	☐	☐	☐	☐
D3. Males have more addiction problems than females	☐	☐	☐	☐	☐
D4. Cannabis use has been associated with an increased risk for deaths	☐	☐	☐	☐	☐
D5. Cannabis use has been associated with an increased risk for psychosis	☐	☐	☐	☐	☐
D6. Patients with drug dependence have frequently othermental disorders	☐	☐	☐	☐	☐
D7. Patients with drug dependence are at increased risk for medical disorders	☐	☐	☐	☐	☐
D8. Patients with drug dependence tend to be poly-abusers	☐	☐	☐	☐	☐
D9. Opioid withdrawal syndrome can be fatal	☐	☐	☐	☐	☐
D10. Alcohol withdrawal syndrome can be managed with benzodiazepines	☐	☐	☐	☐	☐
D11. Delirium tremens is a potentially fatal condition	☐	☐	☐	☐	☐
D12. Psychotherapy should be considered when handling with patients with addiction	☐	☐	☐	☐	☐
D13. Pharmacological treatment should be considered when handling with patients with addiction	☐	☐	☐	☐	☐
D14. Psychosocial interventions should be considered when handling with patients with addiction	☐	☐	☐	☐	☐
D15. Opioid agonist maintenance therapy does not reduces drug use	☐	☐	☐	☐	☐
D16. Methadone should be considered essential medicine for opioid withdrawal/maintenance treatment	☐	☐	☐	☐	☐
D17. Buprenorphine should be considered an essential medicine for both opioid withdrawal and maintenance treatment	☐	☐	☐	☐	☐
D18. Cessation of opioid agonist maintenance treatment is not associated with a high risk of relapse and overdose	☐	☐	☐	☐	☐
D19. Opioid agonist maintenance treatment cannot be continued in long-term	☐	☐	☐	☐	☐
D20. Opioid agonist maintenance therapy does not reduce criminal activity	☐	☐	☐	☐	☐
D21. Opioid agonist maintenance therapy reduces the risk of contracting HIV	☐	☐	☐	☐	☐

D22. Buprenorphine must be administered-taken every day ☐ Yes ☐ No ☐ I don’t know

D23. Methadone must be administered-taken every two-days ☐ Yes ☐ No ☐ I don’t know

D24. Overdose of methadone is usually safer than overdose of buprenorphine ☐ Yes ☐ No ☐ I don’t know

D25. Opioid substitution treatment cannot be used in dependent individuals who are pregnant or breast- feeding ☐ Yes ☐ No

D26. Intake of alcohol and disulfiram together can result in fatal intoxication ☐ Yes ☐ No

D27. Acamprosate has been proven to be effective in reducing relapse for: [Tick only one answer]
☐ alcohol dependence ☐ nicotine dependence ☐ heroin dependence

D28. Naltrexone has been proven to be effective in reducing relapse for: [Tick only one answer]
☐ alcohol dependence ☐ nicotine dependence ☐ methamphetamine dependence

D29. Naloxone is effective in the treatment of: [Tick only one answer]
☐ benzodiazepine overdose ☐ alcohol overdose ☐ opioid overdose

D30. Flumazenil is effective for: [Tick only one answer]
☐ benzodiazepine overdose ☐ alcohol overdose ☐ opioid overdose

D31. The average maintenance daily dose for buprenorphine should be at least: [Tick only one answer]
☐ 3 mg ☐ 6 mg ☐ 8 mg

D32. The average maintenance daily dose for methadone ranges: [Tick only one answer]
☐ from 20 to 40 mg ☐ from 40 to 80 mg ☐ from 60 mg to 120 mg

D33. Have you ever heard about legal highs/ smart drugs/novel substances? ☐ Yes ☐ No

<u>If D33=yes, please fill in also the following questions</u> (D34-D39):

D34. In your understanding, what are common legal highs in your area/country? [you can tick more answers]
☐ 'Salvia'/ Salvia Divinorum
☐ 'Spice drugs' / Synthetic cannabinoids
☐ Bath Salts/ Synthetic cathinones, Mephedrone , MDPV
☐ Kratom / Mitragyna Speciosa
☐ Bromo Dragonfly (B-fly)
☐ Methoxetamine (MXE), Diphenidine (DND), Methoxphenidine (MXP)
☐ NBOMes (N-bombs)
☐ I don't know
☐ Others. Please specify:…………………………………………….

D35. How would you consider your understanding/knowledge of 'legal highs'/'smart drugs'/'novel substances'?
☐ Poor ☐ Basic ☐ Good ☐ Very good

D36. Have you ever seen a patient
Using 'legal highs'/'smart drugs'/novel substances? ☐ Yes ☐ No ☐ I don't know

**PLEASE TICK *ONE* BOX ON EACH LINE**	**Agree strongly**	**Agree**	**Neither agree nor disagree**	**Disagree**	**Disagree strongly**
D37. Legal highs are safer than other drugs for health because are natural and legal	☐	☐	☐	☐	☐
D38. Legal highs are easily controlled and relatively well studied	☐	☐	☐	☐	☐
D39. You can detect patient's legal high use with urine drug screening test	☐	☐	☐	☐	☐
